# Fetal Programming Effects of Testosterone on the Reward System and Behavioral Approach Tendencies in Humans

**DOI:** 10.1016/j.biopsych.2012.05.027

**Published:** 2012-11-15

**Authors:** Michael V. Lombardo, Emma Ashwin, Bonnie Auyeung, Bhismadev Chakrabarti, Meng-Chuan Lai, Kevin Taylor, Gerald Hackett, Edward T. Bullmore, Simon Baron-Cohen

**Affiliations:** a Autism Research Centre, Department of Psychiatry, University of Cambridge, Cambridge, United Kingdom; b Brain Mapping Unit, Department of Psychiatry, University of Cambridge, Cambridge, United Kingdom; c Department of Clinical Biochemistry, Addenbrooke's Hospital, Cambridge, United Kingdom; d Department of Fetal Medicine, Rosie Maternity Hospital, Cambridge, United Kingdom; e Department of Psychology, University of Bath, Reading, United Kingdom; f Centre for Integrative Neuroscience and Neurodynamics, School of Psychology and Clinical Language Sciences, University of Reading, Reading, United Kingdom

**Keywords:** Approach behavior, emotion, fetal programming, fMRI, reward, testosterone

## Abstract

**Background:**

Sex differences are present in many neuropsychiatric conditions that affect emotion and approach-avoidance behavior. One potential mechanism underlying such observations is testosterone in early development. Although much is known about the effects of testosterone in adolescence and adulthood, little is known in humans about how testosterone in fetal development influences later neural sensitivity to valenced facial cues and approach-avoidance behavioral tendencies.

**Methods:**

With functional magnetic resonance imaging we scanned 25 8–11-year-old children while viewing happy, fear, neutral, or scrambled faces. Fetal testosterone (FT) was measured via amniotic fluid sampled between 13 and 20 weeks gestation. Behavioral approach-avoidance tendencies were measured via parental report on the Sensitivity to Punishment and Sensitivity to Rewards questionnaire.

**Results:**

Increasing FT predicted enhanced selectivity for positive compared with negatively valenced facial cues in reward-related regions such as caudate, putamen, and nucleus accumbens but not the amygdala. Statistical mediation analyses showed that increasing FT predicts increased behavioral approach tendencies by biasing caudate, putamen, and nucleus accumbens but not amygdala to be more responsive to positive compared with negatively valenced cues. In contrast, FT was not predictive of behavioral avoidance tendencies, either through direct or neurally mediated paths.

**Conclusions:**

This work suggests that testosterone in humans acts as a fetal programming mechanism on the reward system and influences behavioral approach tendencies later in life. As a mechanism influencing atypical development, FT might be important across a range of neuropsychiatric conditions that asymmetrically affect the sexes, the reward system, emotion processing, and approach behavior.

Many neuropsychiatric conditions affecting emotion processing and approach-avoidance behavioral tendencies (e.g., conduct disorder, psychopathy, attention-deficit/hyperactivity disorder, substance abuse, depression, bipolar disorder, cluster B personality disorders, intermittent explosive disorder, autism) (1) show sex differences in age of onset, risk, prevalence, and symptomatology (2–10). It is also noteworthy that developmental time periods for critical sex steroid surges co-occur with time periods where vulnerability for many of these conditions is elevated (e.g., adolescence), suggesting that mechanisms related to sexual differentiation might play a unique role (11). However, much more work is needed to understand how underlying developmental biological mechanisms related to sexual differentiation (e.g., sex chromosome or sex hormone effects) (12) might help to explain sex differences in these conditions. Unlike work in nonhuman species (13), it is not possible in humans to ethically and independently manipulate factors related to both sex chromosomes and sex hormones within a single study. Thus, in humans it is necessary to focus on each factor separately. In this study we focus on the role of testosterone during fetal development as one developmental biological mechanism that might influence phenotypic development in directions that might increase susceptibility for various neuropsychiatric conditions that asymmetrically affect the sexes.

In adolescence and adulthood, testosterone might increase susceptibility for such neuropsychiatric conditions by tipping the balance between approach and avoidance (14). For example, testosterone in adulthood decreases avoidance by attenuating unconscious fear-responses (15–17) and reducing sensitivity to punishment (18). Similar effects are found in adolescents (19). However, testosterone also increases sensitivity to approach-cues by enhancing attention to social threat (20–22), sensation seeking, motivation to act (23–25), and risk-taking and sensitivity to rewards (18,26). Functional magnetic resonance imaging (fMRI) studies in adolescence and adulthood mirror these effects. Testosterone reduces amygdala response to quick presentations of threat (27) but increases response to longer presentations of threat (28–31) and also increases ventral striatal (e.g., ventral caudate, nucleus accumbens, putamen) response to reward (32–34). Thus, in later life testosterone creates an imbalance between approach and avoidance. However, it is still unclear whether testosterone earlier in development plays a critical prior role in influencing approach-avoidance in brain and behavior.

Despite these later influences of testosterone, work in nonhuman species has shown that early developmental surges should be considered. Early androgens surges can exert “organizational” influence on brain development by laying down permanent cellular and molecular foundations that are necessary for later expression of sex differences (35–39). This idea is similar to the more general idea of fetal programming, which suggests that early events in prenatal development permanently influence developmental paths and outcomes in later life (40). Steroid hormones are well-positioned as fetal programming mechanisms, because they exert substantial epigenetic influence on early brain development that lay the foundations for later interaction with the genome and environmental influences to create variation in neural and behavioral phenotypes (41–44). Thus, understanding the prior influence of testosterone on human brain development is important for understanding how it might program neural circuitry for biased responsiveness later in life and potentially lead individuals down multiple atypical developmental paths.

Here we present the first investigation in humans of how testosterone during fetal development predicts later neural response to valenced facial cues and individual differences in behavioral approach-avoidance tendencies. We tested a unique cohort of 25 boys (8–11 years) whose fetal testosterone (FT) was measured from amniotic fluid at 13–20 weeks gestation. Participants were scanned with fMRI while viewing negative (fear), positive (happy), neutral, or scrambled faces. This paradigm is known to elicit response in both amygdala and ventral striatum (45), which are also known to be influenced by testosterone (27–34). We predicted that increases in FT would predict decreased reactivity to negatively valenced facial cues and increased reactivity to positively valenced facial cues within amygdala and striatum. Furthermore, we predicted that such FT-mediated influence on neural response would predict behavioral approach-avoidance tendencies.

## Methods and Materials

### Participants

Participants were recruited as part of a longitudinal study of the effects of FT on cognitive, behavioral, and brain development. Initial screening consisted of reviewing medical records of patients who underwent amniocentesis in the Cambridgeshire (United Kingdom) region. Individuals were excluded if: 1) the amniocentesis revealed a chromosomal abnormality; 2) there was a twin pregnancy; 3) the pregnancy ended in termination or miscarriage; 4) relevant information was absent from the medical records; or 5) medical practitioners indicated it would be inappropriate to contact the family. Any child that presented with any developmental abnormalities postnatally was also excluded from testing. Twenty-eight right-handed typically developing boys were successfully scanned, but 3 were excluded for excessive motion, leaving 25 for the main analyses (mean age = 9.52, SD = .96, range: 8–11 years). Our use of a male-only sample was employed to eliminate potential nonhormonal confounds that systematically vary across male and female subjects (e.g., sex chromosome effects) (12). Informed consent was obtained from all the legal guardians of the participant in accordance with procedures approved by the local research ethics committee. This cohort has been previously reported in published works examining the relationship between FT and structural magnetic resonance imaging measures (38,46).

### FT Collection and Measurement

Fetal testosterone was measured from amniotic fluid samples collected between 13 and 20 weeks of gestation (mean = .76 nmol/L, SD = .35 nmol/L, range = .25–1.70 nmol/L). This is within the 8–24-week period that is critical for human sexual differentiation (47). Six participants from the sample had missing data with regard to the exact time of amniocentesis. However, analysis of the remaining 19 participants (mean = 16.37, SD = 1.33, range = 14–19) showed that there was no relationship between gestational age at sampling and FT level (*r* = .17, *p* = .47), confirming prior work showing absence of a relationship (48). Thus, all 25 participants were included in the final analyses.

FT was assayed via radioimmunoassay. Amniotic fluid was extracted with diethyl ether, which was evaporated to dryness at room temperature, and the extracted material was re-dissolved in an assay buffer. Testosterone was assayed by the DPC ‘Count-a-Coat' method (Diagnostic Product Corporation, Los Angeles, California), which uses an antibody to testosterone coated onto propylene tubes and a 125-I labeled testosterone analogue. The detection limit of the assay with the ether-extraction method is approximately .05 nmol/L. The coefficient of variation (CV) for between-batch imprecision is 19% at a concentration of .8 nmol/L and 9.5% at a concentration of 7.3 nmol/L. The CVs for within-batch imprecision are 15% at a concentration of .3 nmol/L and 5.9% at a concentration of 2.5 nmol/L. This method measures total extractable testosterone.

### Task Design and Behavioral Measures

Participants were scanned while viewing fear, happy, neutral, and scrambled faces taken from the standard Karolinska Directed Emotional Faces set (49). Specific stimuli were chosen from the Karolinska Directed Emotional Faces on the basis of unanimous ratings of the target expression from five independent judges.

For each trial a face was presented on the screen for 2000 msec, followed by a central crosshair for 750 msec, followed by an intertrial interval of 312 msec before the onset of the next trial. Conditions were presented in separate blocks, with 8 trials/block. Each block lasted 24.5 sec and was repeated four times in pseudorandom order. Throughout the experiment, participants were instructed to press a button with their right index finger whenever a face was presented. Stimulus presentation was implemented with DMDX software (http://www.u.arizona.edu/∼kforster/dmdx/dmdx.htm), and stimulus presentation was synchronized with the onset of the functional run to ensure accuracy of event timing.

Individual differences in behavioral approach-avoidance tendencies were assessed by caregiver report on a modified version of the Sensitivity to Punishment and Sensitivity to Rewards questionnaire (50). Previous factor analyses suggest that the Sensitivity to Punishment and Sensitivity to Rewards questionnaire can be split into 4 subscales: Punishment, Impulsivity/Fun-Seeking, Drive, and Reward Responsivity. The Punishment scale was used as our measure of avoidance tendencies and was termed “BIS” after Gray's “behavioral inhibition system” (51). For behavioral approach tendencies, we created a summary score by summing across all items on the other three scales (Impulsivity/Fun-Seeking, Drive, and Reward Responsivity). We term this summary score “BAS” after Gray's “behavioral activation system” or “behavioral approach system” (51).

### fMRI Data Acquisition

All imaging took place at the Wolfson Brain Imaging Centre at Addenbrooke's Hospital, Cambridge, United Kingdom, on a Siemens Tim Trio 3 Tesla magnet (Siemens Medical Solutions, AG, Erlangen, Germany). Our functional imaging run consisted of 200 whole-brain functional T2*-weighted echoplanar images (slice thickness, 3 mm; .75 mm skip; 32 axial slices; repetition time, 2000 msec; echo time, 30 msec; flip angle, 90°; matrix, 64 × 64; field of view, 192 mm; interleaved slice acquisition). The first six timepoints of the run were discarded to allow for T2 stabilization effects. In addition, a high-resolution T1-weighted three-dimensional magnetization-prepared rapid acquisition gradient-echo (MP-RAGE) structural image was acquired for registration purposes (slice thickness = 1 mm; repetition time = 2300 msec; echo time = 2.98 msec; field of view = 256 × 240 × 176 mm; flip angle = 9°; voxel size = 1 mm^3^ isotropic).

### Data Analysis

fMRI data preprocessing and first-level statistics were implemented in SPM8 (http://www.fil.ion.ucl.ac.uk/spm). The preprocessing steps were conducted in the following manner: functional data were slice-timing corrected and realigned to the mean functional image. Next, the realigned and slice-timing corrected functional data were co-registered to the high-resolution MP-RAGE. The high-resolution MP-RAGE was then segmented into cerebrospinal fluid and gray and white matter, with prior tissue probability maps for this step generated from the Template-O-Matic toolbox (http://dbm.neuro.uni-jena.de/software/tom/) (52), set for the age range of 8–11 years. The normalization transformation matrix from the segmentation step was then applied to the functional and structural images, thus transforming it into standard anatomical space on the basis of the ICBM 152 brain template (Montreal Neurological Institute) at a resolution of 2 mm (isotropic) voxels. Smoothing was applied at 4 mm full-width-at-half-maximum (FWHM), to retain sensitivity for smaller regions such as the amygdala and ventral striatum.

First-level analyses were performed with the general linear model in SPM8. Each trial was convolved with the canonical hemodynamic response function. High-pass temporal filtering with a cutoff of 128 sec was applied to remove low-frequency drift in the time-series, and global changes were removed by proportional linear scaling. Serial autocorrelations were estimated with a restricted maximum likelihood algorithm with an autoregressive model of order 1. Five contrasts from the first-level analyses were used in subsequent second-level analyses. Here we compared fear or happy faces against two high-level control conditions of neutral faces or scrambled faces (i.e., Fear > Neutral, Fear > Scrambled, Happy > Neutral, Happy > Scrambled). Last we compared happy against fear (Happy > Fear) as a direct contrast of valenced facial cues. These contrast images were input into second-level whole-brain random-effects analyses. Chronological age was used as a covariate, and FT was the predictor variable of interest. Given our a priori interest in the striatum and amygdala, we reduced the search space for second-level analyses with an explicit mask combining the caudate, putamen, nucleus accumbens, and amygdala defined by the Harvard-Oxford subcortical atlas in FSL (http://www.fmrib.ox.ac.uk/fsl/). Analyses were thresholded with a cluster-forming height threshold of *p* < .025 and topological FDR control for multiple comparison correction at *q* < .05 (53).

Next, we used statistical mediation analyses to test whether FT predicts behavioral approach or avoidance tendencies via influence on striatum and amygdala. Here we used anatomically defined regions of interest (ROIs) of caudate, putamen, nucleus accumbens, and amygdala from the Harvard-Oxford subcortical atlas in FSL (http://www.fmrib.ox.ac.uk/fsl/) to extract mean percent signal change from each ROI for the valence contrast of Happy > Fear. In these analyses, the predictor variable was FT, the outcome variable was either BAS or BIS, and the mediator was Happy > Fear ROI response. Chronological age was included as a covariate. For statistical inference we used bootstrapping (100,000 resamples) to estimate whether 0 was within or outside the 95% bias-corrected and accelerated bootstrap confidence intervals. All mediation analyses were implemented in the M3 Mediation Toolbox (http://wagerlab.colorado.edu/files/tools/mediation.html) (54) in Matlab 7.11.

## Results

### Behavioral Data

**Figure 1:**
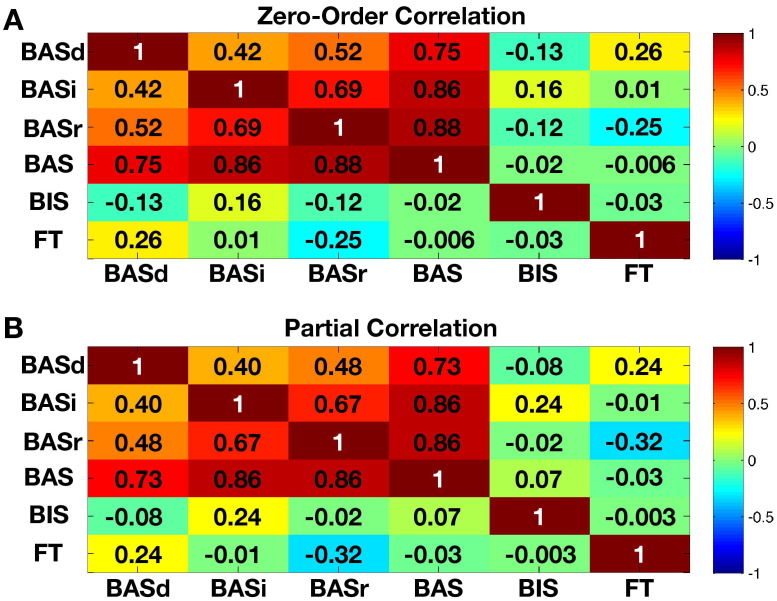
Correlations between behavioral approach system (BAS) score across all subscales, behavioral inhibition system (BIS) punishment subscale, and fetal testosterone (FT). This figure presents correlation matrices representing **(A)** zero-order correlations and **(B)** partial correlations after partialing out chronological age across FT, BIS, and all BAS subscales. BASd, BAS drive subscale; BASi, BAS impulsivity/fun-seeking subscale; BASr, BAS reward responsivity subscale.

FT was not significantly related to BIS (i.e., Punishment) (*r* = −.03, *p* = .89), Impulsivity/Fun-Seeking (*r* = .01, *p* = .96), Drive (*r* = .26, *p* = .26), or Reward Responsivity (*r* = −.25, *p* = .27) subscales. These correlations remained nonsignificant after partialing out age. FT also did not correlate with total BAS score (i.e., summing across Drive, Impulsivity/Fun-Seeking, and Reward Responsivity subscales) (*r* = −.0006, *p* = .99). Finally, although BIS and BAS were not correlated with each other (*r* = −.02, *p* = .93; after partialing out age: *r* = .07, *p* = .77), all BAS subscales were highly correlated with each other (all *r* > .40, all *p* < .05). See Figure 1 for correlation matrices.

### fMRI Data

**Figure 2:**
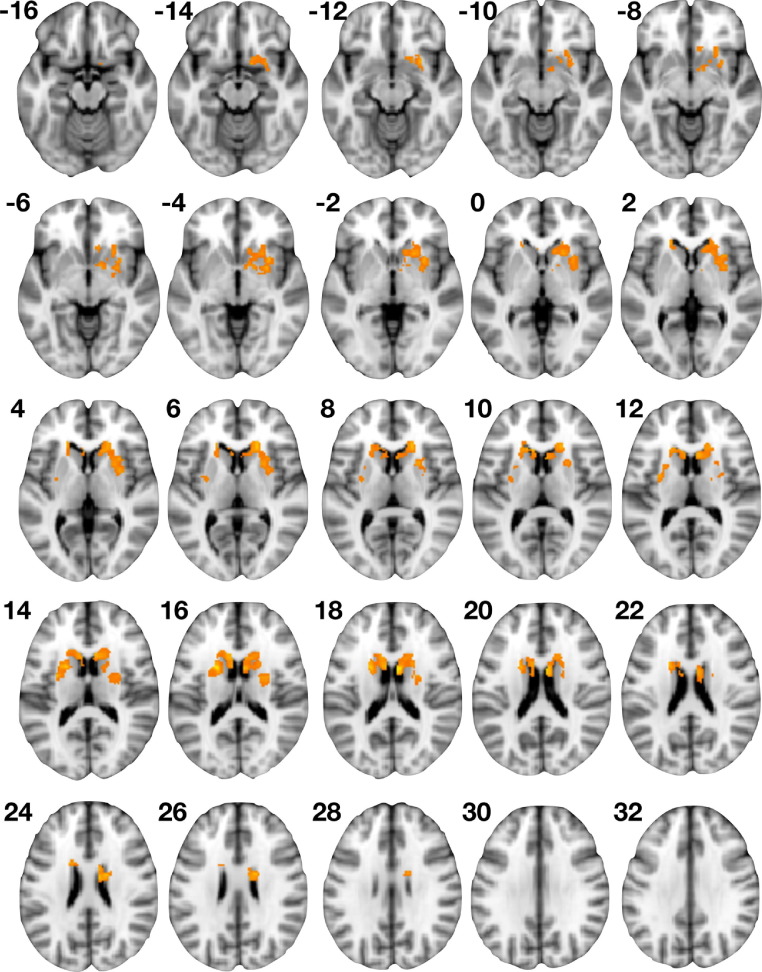
Association between neural response to valenced information (Happy > Fear) and fetal testosterone (FT). This figure shows areas within the striatum and amygdala where Happy > Fear activation is positively correlated with FT. Numbers indicate *z*-slice coordinate in Montreal Neurological Institute space.

No regions significantly correlated with FT across the contrasts of Fear > Neutral, Fear > Scrambled, Happy > Neutral, and Happy > Scrambled. However, for Happy > Fear there were two bilateral clusters showing a positive correlation with FT. The left hemisphere cluster comprised voxels within the dorsal portions of the caudate nucleus and putamen but did not include voxels within nucleus accumbens or amygdala. The right hemisphere cluster comprised voxels across caudate, putamen, and nucleus accumbens but not amygdala (Figure 2, Table 1). For results of activation analyses on all contrasts irrespective of FT, see Table S1 in Supplement 1.

Next, we used statistical mediation analyses to test the hypothesis that FT predicts approach or avoidance behavioral tendencies via its influence on neural response to valenced facial cues (i.e., Happy > Fear) within bilateral striatal or amygdala ROIs. The relationship between FT (i.e., predictor) and Happy > Fear ROI response (i.e., mediator) is noted as “path a.” The relationship between Happy > Fear ROI response and outcome variable (BIS or BAS), controlling for the influence of FT, is noted as “path b.” The overall FT-outcome relationship (ignoring the mediator) is the total effect or “path c,” while the direct effect of FT on outcome variable, controlling for Happy > Fear ROI response is noted as “path c'.” Finally, the “mediation effect” is noted as “a*b” and tests whether the difference between path c and path c' is significantly different from zero. In other words, the mediation effect tests whether the inclusion of the mediator (i.e., Happy > Fear ROI response) accounts for a significant amount of variance in the relationship between FT and outcome variable, compared with the total relationship between FT and outcome variable when the mediator is not taken into account.

**Figure 3:**
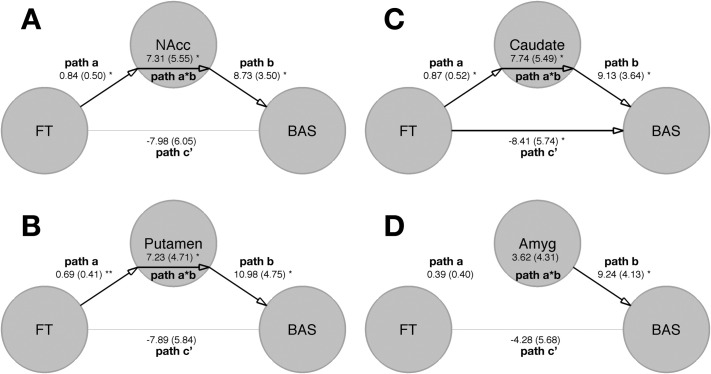
Path diagrams of relationships between fetal testosterone (FT), neural mediators in the striatum or amygdala, and total behavioral approach system (BAS) score summing across all subscales. **(A)** Path diagram when nucleus accumbens (NAcc) Happy > Fear response is the mediator between FT and BAS. **(B)** Path diagram when putamen Happy > Fear response is the mediator between FT and BAS. **(C)** Path diagram when caudate Happy > Fear response is the mediator between FT and BAS. **(D)** Path diagram when amygdala (Amyg) Happy > Fear response is the mediator between FT and BAS. Path a is the relationship between the predictor (FT) and the mediator (region of interest [ROI]). Path b is the relationship between the mediator (ROI) and the outcome (BAS), controlling for the predictor (FT). Path c' is the relationship between the predictor (FT) and the outcome (BAS) controlling for the mediator (ROI). Path c is the total effect of the relationship between the predictor (FT) and the outcome (BAS), irrespective of the mediator. Path a*b is the difference between path c and path c'. Path coefficients and standard errors (in parentheses) are noted for each path. *p < .05, **p < .01.

When BAS was the outcome variable, only striatal ROIs (i.e., caudate, putamen, and nucleus accumbens) showed significant mediation effects (i.e., path a*b). The directionality of path coefficients suggested that such effects can be interpreted as increasing FT influences increases in BAS scores via FT-mediated increases in striatal bias for positive (happy faces) compared with negatively valenced facial cues (fear faces). No mediation effect was observed within the amygdala. However, path b was significant for the amygdala. This suggests that amygdala valence selectivity is positively associated with BAS when controlling for FT-influence on BAS (Figure 3A–D, Table 2). When BIS was the outcome variable, no ROIs showed mediation effects, and no paths incorporating the mediator were significant (i.e., path b, path c') (Table 3).

## Discussion

In this study we investigated whether FT in human male subjects acts as a fetal programming mechanism on the developing brain and behavioral approach-avoidance tendencies. We further tested whether any prior influence of FT on behavioral approach-avoidance tendencies was mediated by FT-influence on the reward system. First, we found no correlation between FT and BIS or BAS (both with and without partialing out age effects). This suggests that, without taking into account any influence FT has on the brain, FT does not influence BIS or BAS. However, fMRI analyses showed that FT was a significant predictor of striatal but not amygdala sensitivity to valenced facial cues. In particular, we only found effects under the direct contrast of valenced facial cues (i.e., Happy > Fear). The restricted nature of this type of FT-influence for the valence contrast—but not when compared with neutral or scrambled faces—might suggest subtle influences on sensitivity to valenced information rather than a more general effect for only one type of valenced information (e.g., only positive or only negative). This point might be particularly important in future work, because the FT-influence might only be pronounced when unambiguously positive and negative information-processing are contrasted against each other.

With statistical mediation analyses we also observed that valence selectivity in striatal nuclei such as caudate, putamen, and nucleus accumbens mediate FT influence on BAS but not BIS. This means that increasing levels of FT predicts later increases in behavioral approach but not avoidance tendencies by organizing the striatum for biased selectivity for positive over negatively valenced information. In contrast to the striatum, no such FT-mediation effects were observed for amygdala when the outcome was BIS or BAS. However, conceptually replicating prior work, we found that amygdala response was positively associated with BAS (55). These results provide important developmental biological insights into individual differences in approach behavior and emotion processing in the general population. Individual differences in how the brain responds to emotion are well-documented. For instance, personality traits modulate the degree to which amygdala and ventral striatum respond to positive or negatively valenced information (56,57). The current results suggest that FT is an early developmental biological mechanism that might explain later emergence of individual differences in biased neural response to valenced information and behavioral approach tendencies.

The mediation results are also important in relation to the development of extremes of emotion processing and approach behavior. Androgens have been hypothesized to be linked to neuropsychiatric conditions that asymmetrically affect the sexes (e.g., conduct disorder, psychopathy, attention-deficit/hyperactivity disorder, substance abuse, depression, bipolar disorder, cluster B personality disorders, intermittent explosive disorder, autism) (1–11,14,18,58–63). Prior work within these conditions shows that ventral striatal abnormalities are common, particularly in adolescence (64–71). Adolescence is a time period for increased vulnerability to many of these conditions (11). Emerging work has shown that the reward system in adolescence is more sensitive than in adulthood (72–74). However, increased sensitivity to reward is reversed in adolescents who were characterized in early childhood as having a behaviorally inhibited temperament (75). Although one mechanism for sensitivity to reward in adolescence could be current testosterone levels (33,34), the current study suggests that events in fetal development might play an earlier organizational role in laying down cellular/molecular foundations in the reward system that allow for enhanced vulnerability in adolescence.

Given the temporal precedence of FT in development, one explanation for current known effects of testosterone on the reward system later in life (32–34) could be because it acts upon prior organized neural circuitry influenced by FT in early brain development. Much precedence for this idea can be found in the nonhuman literature, where experimental control can be exerted over hormones at early and later stages of development (12). In humans, emerging work suggests that similar principles might apply. For example, in two recent studies by van Honk *et al.* (37,39) it was shown that current testosterone levels affected mentalizing ability and social cooperation behavior, but this effect varied as a function of fetal androgens and estrogens as measured by the proxy of 2D:4D digit ratio. Although the current study did not measure current testosterone, it is an open question for future research whether activational effects of testosterone in adolescence place an individual at increased risk for various types of psychopathology because of prior organizational foundations built during fetal development and influenced by FT.

At a theoretical level, several hypotheses can be generated from the current study. Atypical FT levels might be an individual risk factor for various types of psychopathology by exerting early epigenetic influence on the expression of different sets of risk genes (41,43,44). Multiple atypical developmental pathways might be opened by FT-influence on behavioral approach tendencies and reward system sensitivity to approach cues in the environment. FT might also increase susceptibility to atypical environmental or other biological (e.g., later androgen surges, cortisol) risk factors at later points in development. Thus, the current work suggests promise in examining the prior influence of FT on the development of multiple neuropsychiatric phenotypes that asymmetrically affect the sexes.

There are some limitations and caveats for the current study. First, the children in this study were approximately at the cusp of prepuberty to early puberty, but measures of current testosterone were unavailable. One reason for this is because in pilot work we encountered significant difficulty in getting young children of the same age range to produce a sufficient volume of saliva necessary for the assays. However, because chronological age in this particular cohort likely covaries with age of onset of early puberty, older children (i.e., 11 years) will likely be on the cusp of early puberty and have higher current testosterone levels than younger children (i.e., 8 years) and were more likely to be in the pre-pubertal stage and adrenarche. All analyses incorporated chronological age as a covariate, and this likely exerted some statistical control over the unmeasured effect of current testosterone. Furthermore, as past work has shown (33,34), the effects of current testosterone are in the same direction as those documented in this study by FT, leaving the possibility open that any current testosterone effects are dependent on earlier organizational influence of FT (37,39). However, given that residual levels of pubertal testosterone after correcting for age are known to predict individual differences in structural brain development (76,77), future work is necessary for explicitly contrasting any effects of current testosterone with FT.

Second, given that amniocentesis is typically conducted for clinical reasons (e.g., screening for chromosomal abnormalities) on older mothers, the cohort we studied is a selective subsample of the general population. Although all the participants in the current study were considered “typically developing” children, this work requires replication in a more representative sample of the general population. Furthermore, follow-up work on larger datasets where amniocentesis samples are available would benefit from comparing groups of individuals who are considered “typically developing” from those with various types of psychopathology that asymmetrically affect the sexes.

Finally, it should be noted that the measure used for BIS and BAS was a parental-report instrument. Although we included this type of measure to bypass difficulties with self-reports of young children, one caveat to this might be that parents might not be fully aware of the behaviors of their child, especially with regard to adolescence. Given that all the children in this study were likely prepubertal or at the beginning of puberty, parental awareness of the behaviors of their child are relatively less of an issue compared with children in later adolescence. However, future work comparing the correspondence between self-report and parental-reports in relation to FT would be valuable.

In summary, FT in humans plays an important fetal programming influence on the developing reward system and behavioral approach tendencies. Fetal testosterone exerts its influence on behavioral approach tendencies by biasing reward system sensitivity to valenced information. This work not only is relevant to the development of individual differences in the general population but might also be critical for understanding the mechanisms behind a range of neuropsychiatric conditions that asymmetrically affect the sexes, emotion processing, approach behavior, and the reward system (1–10).

## Figures and Tables

**Table 1:** Areas Where Happy > Fear Response Is Positively Associated with FT

Region	MNI (x,y,z)	*t*	Cluster Size	*p* (FDR)
Right Hemisphere			1197	.0001
Caudate	6,4,18	4.92		
Putamen	32,10,−4	3.31		
Nucleus accumbens	12,8,−14	2.89		
Left Hemisphere			467	.02
Caudate	−20,6,16	4.55		
Putamen	−30,−2,14	3.72		

Results from voxel-wise analysis where search space was reduced to anatomically defined areas of the caudate, nucleus accumbens, putamen, and amygdala. Thresholding consisted of a cluster-forming height threshold of *p* < .025 and topological false discovery rate (FDR) cluster-correction at *q* < .05. FT, fetal testosterone; MNI, Montreal Neurological Institute.

**Table 2:** Mediation Analyses Results with BAS as the Outcome Variable

Region	Path a	Path b	Path c'	Path c	Path a*b
Amygdala					
Path coefficient	.39	9.24	−4.28	−.67	3.62
STE	.40	4.13	5.68	6.24	4.31
*p*	.32	.04	.22	.70	.25
Caudate					
Path coefficient	.87	9.13	−8.41	−.66	7.74
STE	.52	3.64	5.74	6.23	5.49
*p*	.01	.01	.04	.70	.02
Nucleus Accumbens					
Path coefficient	.84	8.73	−7.98	−.67	7.31
STE	.50	3.50	6.05	6.15	5.55
*p*	.01	.02	.07	.71	.01
Putamen					
Path coefficient	.69	10.98	−7.89	−.65	7.23
STE	.41	4.75	5.84	6.22	4.71
*p*	.005	.02	.06	.71	.01

Results from mediation analyses where fetal testosterone is the predictor, mean Happy > Fear region of interest response is the mediator, and total behavioral approach system (BAS) score summing across all subscales is the outcome variable. Path a is the relationship between the predictor and the mediator. Path b is the relationship between the mediator and the outcome, controlling for the predictor. Path c' is the relationship between the predictor and the outcome controlling for the mediator. Path c is the total effect of the relationship between the predictor and the outcome, irrespective of the mediator. Path a*b is the difference between path c and path c'. Hypothesis testing and statistical significance was evaluated with bootstrapping. An effect was statistically significant if the value 0 was not within the 95% bias-corrected and accelerated bootstrap confidence intervals. STE, standard error.

**Table 3:** Mediation Analyses Results with BIS as the Outcome Variable

Region	Path a	Path b	Path c'	Path c	Path a*b
Amygdala					
Path coefficient	.38	.92	−1.52	−.84	.67
STE	.39	3.28	5.88	5.02	2.01
*p*	.31	.82	.78	.89	.62
Caudate					
Path coefficient	.87	.50	−1.13	−.85	.28
STE	.52	2.65	5.91	5.03	2.62
*p*	.01	.91	.83	.90	.92
Nucleus Accumbens					
Path coefficient	.84	.68	−1.27	−.82	.45
STE	.51	2.92	6.00	5.01	2.92
*p*	.009	.97	.88	.91	.99
Putamen					
Path coefficient	.69	−.06	−.67	−.83	−.16
STE	.40	3.22	5.69	5.00	2.35
*p*	.005	.90	.93	.90	.89

Results from mediation analyses where fetal testosterone is the predictor, mean Happy > Fear region of interest response is the mediator, and behavioral inhibition system (BIS) punishment subscale is the outcome variable. Path a is the relationship between the predictor and the mediator. Path b is the relationship between the mediator and the outcome, controlling for the predictor. Path c' is the relationship between the predictor and the outcome controlling for the mediator. Path c is the total effect of the relationship between the predictor and the outcome, irrespective of the mediator. Path a*b is the difference between path c and path c'. Hypothesis testing and statistical significance was evaluated with bootstrapping. An effect was statistically significant if the value 0 was not within the 95% bias-corrected and accelerated bootstrap confidence intervals. STE, standard error.
