# Supplementary material for: Fetal Programming Effects of Testosterone on the Reward System and Behavioral Approach Tendencies in Humans
Source: Biol Psychiatry. 2012 Nov 15;72(10):839–47. doi: 10.1016/j.biopsych.2012.05.027 (PMC3485553; doi:10.1016/j.biopsych.2012.05.027)
Supplement: Supplement 1 [file mmc1.pdf]

Fetal Programming Effects of Testosterone on the Reward System and Behavioral Approach Tendencies in Humans

Supplemental Information

Results from Analyses of Activation on the Main Effects Contrasts

Using the same anatomical regions of interest (ROIs) for the fetal testosterone (FT) analysis, we tested for activations at either a cluster-level of correction or at a voxel-level (using small-volume correction for specific ROIs). For the Fear>Scrambled contrast, there were 2 significant activations at false discovery rate (FDR) cluster-corrected levels. Within right-hemisphere the peak for the cluster was located in the amygdala (x = 24, y = -2, z = -16, k = 2185, t = 5.63, $p = 2.01 \times 10^{-6}$) and the cluster extended across putamen, nucleus accumbens and caudate. Within left-hemisphere the peak for the cluster was located in the putamen (x = -24, y = 12, z = 2, k = 1362, t = 5.28, $p = 1.41 \times 10^{-4}$) and the cluster extended across amygdala and nucleus accumbens, but did not include the caudate. Across all other contrasts, no significant activations were found at FDR cluster-corrected levels. We next used small-volume correction within each specific ROI. A summary of these results can be found in Table S1. Across all contrasts except Happy>Fear, the amygdala was significantly activated at or above trend levels of significance. However, the striatal sub-nuclei were not activated consistently across all contrasts, with putamen only reaching significance for Fear>Scrambled. Given that there are substantial individual differences in neural systems for emotion processing (1, 2), we view these results as being secondary to the main results which highlight FT as one individual difference variable that accounts for substantial variation in neural response to emotion.

Table S1. Activation analysis results for all contrasts. All results are reported after small-volume correction.

Region	Hemisphere	MNI (x, y, z)	*t*-value	*p*-value (FWE)
**Fear > Neutral**				
Caudate	L	-12, 6, 10	2.59	0.87
Caudate	R	22, 16, -6	2.70	0.83
Putamen	L	22, -2, -10	4.15	0.16
Putamen	R	-18, 0, -10	2.98	0.74
Nucleus Accumbens	L	18, 14, -8	2.44	0.55
Nucleus Accumbens	R	-16, 10, -8	2.62	0.46
Amygdala	L	-16, -6, -12	3.79	0.085
Amygdala	R	22, -2, -10	4.25	0.044
**Fear > Scrambled**				
Caudate	L	20, 8, 8	4.68	0.06
Caudate	R	-14, -20, 18	3.53	0.41
Putamen	L	-24, 12, 2	5.28	0.02
Putamen	R	22, 8, 8	4.97	0.04
Nucleus Accumbens	L	-10, 22, -4	3.46	0.15
Nucleus Accumbens	R	16, 6, -8	3.92	0.07
Amygdala	L	-18, -8, -14	4.09	0.056
Amygdala	R	24, -2, -16	5.63	0.003
**Happy > Neutral**				
Caudate	L	-12, -16, 24	2.02	0.98
Caudate	R	14, -18, 24	2.16	0.97
Putamen	L	-22, -2, -10	2.62	0.91
Putamen	R	22, -2, -10	3.22	0.62
Nucleus Accumbens	L	-16, 10, -8	1.57	0.88
Nucleus Accumbens	R	10, 4, -14	1.52	0.90
Amygdala	L	-18, -4, -12	4.03	0.058
Amygdala	R	32, 4, -16	4.54	0.022
**Happy > Scrambled**				
Caudate	L	-20, -14, 26	2.64	0.57
Caudate	R	16, -4, 16	3.84	0.28
Putamen	L	-22, 0, -10	3.71	0.38
Putamen	R	28, -12, 8	3.71	0.38
Nucleus Accumbens	L	-4, 4, -12	3.70	0.11
Nucleus Accumbens	R	6, 8, -14	3.36	0.19
Amygdala	L	-18, -10, -12	5.36	0.005

Amygdala	R	18, -10, -12	5.34	0.005
**Happy > Fear**				
Caudate	L	-18, -4, -16	1.79	0.88
Caudate	R	18, 2, 20	1.89	0.86
Putamen	L	-24, 0, 0	0.74	0.99
Putamen	R	30, 18, -2	1.69	0.99
Nucleus Accumbens	L	-2, 14, -2	1.67	0.89
Nucleus Accumbens	R	2, 12, -2	1.39	0.94
Amygdala	L	-18, -4, -16	1.79	0.88
Amygdala	R	18, 2, -20	1.89	0.86

FWE, family-wise error; L, left; MNI, Montreal Neurological Institute; R, right.

Supplemental References

1. Calder AJ, Ewbank M, Passamonti L (2011): Personality influences the neural responses to viewing facial expressions of emotion. *Philos Trans R Soc Lond B Biol Sci*. 366:1684-1701.
2. Hamann S, Canli T (2004): Individual differences in emotion processing. *Curr Opin Neurobiol*. 14:233-238.
